# Body Composition and Incident High‐Intensity Back Pain and/or High Disability: A 10‐Year Prospective Population‐Based Male Cohort

**DOI:** 10.1002/jcsm.13641

**Published:** 2024-11-24

**Authors:** Mahnuma Mahfuz Estee, Yuanyuan Wang, Stephane Heritier, Donna M. Urquhart, Flavia M. Cicuttini, Mark A. Kotowicz, Sharon L. Brennan‐Olsen, Julie A. Pasco, Anita E. Wluka

**Affiliations:** ^1^ School of Public Health and Preventive Medicine Monash University Melbourne Victoria Australia; ^2^ IMPACT—Institute for Mental and Physical Health and Clinical Translation Deakin University Geelong Victoria Australia; ^3^ Department of Medicine–Western Health University of Melbourne St Albans Victoria Australia; ^4^ University Hospital Geelong Barwon Health Geelong Victoria Australia; ^5^ School of Health and Social Development Deakin University Geelong Victoria Australia; ^6^ Australian Institute for Musculoskeletal Sciences (AIMSS), Western Health University of Melbourne St Albans Victoria Australia

**Keywords:** back disability, back pain, body composition, body mass index, lean mass, weight gain

## Abstract

**Background:**

Back pain poses a significant global burden, within which individuals with more severe symptoms consume higher healthcare expenses than those with lesser back pain. Whether measures of body composition predict high‐intensity back pain and/or high‐disability in population‐based cohorts is unknown. This study aimed to examine the association between body composition at baseline and their change in the prior 5 years (between 2001–2005 and 2006–2010) and incident high‐intensity back pain and/or high‐disability in long‐term follow‐up, 10 years later (2016–2021) in a population‐based cohort of men.

**Method:**

This study examined men with no or low‐intensity back pain and disability (Graded Chronic Pain Scale) at back pain study baseline (2006–2010) within the Geelong Osteoporosis Study. Those developing high‐intensity pain and/or high disability at follow‐up (2016–2021) were identified. Weight, body mass index (BMI), abdominal circumferences, fat mass and lean mass (dual energy X‐ray absorptiometry) were assessed prebaseline (2001–2005) and at baseline. The association of body composition at baseline and change in body composition from prebaseline to baseline with incident high‐intensity pain and/or high disability at follow‐up were examined using multivariable logistic regression.

**Result:**

Of 695 participants with no or low‐intensity pain and disability at baseline, 441 (62.3%) completed follow‐up with a mean age of 54.3 ± 14.1 years: 37 (8.3%) developed high‐intensity pain and/or high‐disability, 33 (7.5%) developed high‐intensity pain and 14 (3.2%) high disability. No measures of body composition at baseline were associated with incident high‐intensity pain and/or high disability at follow‐up in the whole population. In subgroup analysis, among men aged over 60 years, but not younger, higher lean mass was associated with decreased likelihood of high‐intensity pain and/or high‐disability (odds ratio [OR] 0.86, 95% confidence interval [CI] 0.76, 0.97, interaction *p* < 0.001). In the whole population, examination of the relationship between change in measures of body composition between prebaseline and baseline, only a one unit increase in BMI, equivalent to 3.1‐kg weight gain, was associated with increased incident high disability (OR 1.63, 95% CI 1.06, 2.51).

**Conclusion:**

In a population‐based sample, without severe back pain and disability, in older men aged ≥60 years, higher lean mass was protective of incident high‐intensity pain and/or high disability. An increase in BMI, over 5 years, equivalent to 3.1‐kg weight gain, was associated with incident back pain related high disability 10 years later. These results demonstrate another detrimental consequence of weight gain and highlight the importance of maintaining muscle mass in older men.

## 1 Introduction

Global public health interest in back pain is increasing, with the rising prevalence and burden of the disease [1]. Back pain was estimated to affect 619 million people in 2020, rising to 850 billion by 2050 [1]. Back pain and related disability limit involvement in regular work, physical activity, socialisation and personal care [2] and cost 4.8 billion AUD annually in Australia [3]. Although low‐intensity back pain may fluctuate, tending to recover, high‐intensity pain tends to be more persistent [4]. Individuals with severe symptoms of back pain consume double the healthcare expenses used by those with low‐impact pain and are responsible for more than three fourths of the year lived with disability attributed to back pain [5]. Moreover, high disability is associated with 2.5 times higher healthcare cost or societal cost and poorer quality of life than those with only pain [6]. Nevertheless, there are limited effective treatments for back pain and disability [7]. Thus, there is an urgent need to identify modifiable factors to be targeted in order to reduce the burden imposed by high‐intensity back pain and related disability.

The prevalence of back pain is higher in women compared with men [8], attributing to anatomical, biological, psychological and sociocultural factors that differ in men and women [9, 10]. However, in men of the working age group (45–74 years), the prevalence of back problems including back pain is higher in Australia [11]. The duration of back pain related absence from work was higher in men (median 12 days) compared with women (median 7 days) in a 1‐year prospective population‐based cohort study [12]. Thus, predictive factors for back pain need to be examined in men and women separately. Identification of gender specific predictive factors may enable health professionals to organise personalised management for back pain. However, few studies have examined risk factors for back pain in men.

The association between body composition and back pain has not been established in a population‐based sample of men. In general, obesity poses a risk for back pain by direct mechanical load and chronic metabolic inflammation [13]. However, results of studies of the longitudinal associations between BMI and back pain in population‐based studies in men have been inconsistent [14, 15, 16, 17]. Similarly, the relationships identified between higher body fat mass and lower lean mass with back pain in men from cross‐sectional/case–control studies vary [18, 19, 20, 21]. Thus, the relationship between measures of body composition and incident back pain is uncertain. Previous studies have reported inconsistent results that may be explained by the failure to consider measures of body composition and incident high‐intensity back pain and/or high‐disability in community‐based men, spanning the full age range, over the long term. Although only one study examined association between change in BMI and subsequent risk of back pain, a significant association was found between an increase in BMI and the risk for chronic back pain in adults [22]. However, this study did not perform analysis in men separately. Thus, whether changes in these measures of body composition over 5 years have any impact on incident high‐intensity back pain and/or high‐disability in later life in population‐based men is unknown.

Therefore, the aims of the current study were (i) to examine the long‐term (over 10 years) associations between body composition at 2006–2010 and incident high‐intensity back pain and/or high disability in population‐based men and (ii) to examine whether changes in body composition between 2001–2005 and 2006–2010 predicts incident high‐intensity back pain and/or high disability after 10 years in a population‐based cohort of men.

## 2 Method

### 2.1 Study Population

**FIGURE 1:**
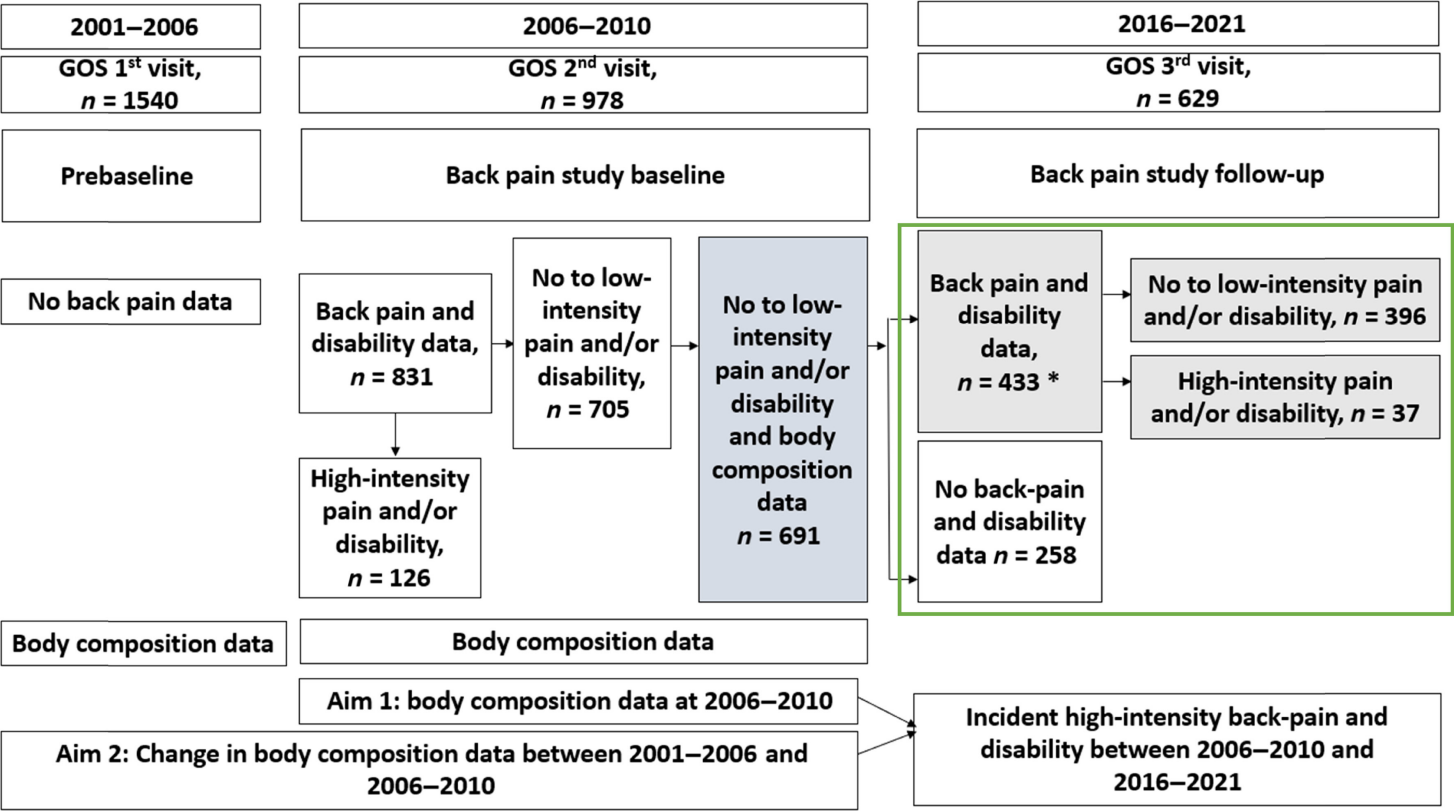
Flow chart showing the number of participants in the Geelong Osteoporosis Study (GOS) and the current back pain study (green box). *435 participants provided back pain data and 433 participants back pain related disability data at back pain study follow‐up. Body composition (weight, body mass index, waist circumference, hip circumference, fat mass and lean mass) were collected at back pain study baseline and 5 years prior to baseline. Change in these measures in the preceding 5 years was calculated by subtracting GOS 1st visit data from GOS 2nd visit data. Associations of body composition at baseline or change in these measures in the preceding 5 years and incident high‐intensity pain and/or high disability after 10 years were evaluated.

The Geelong Osteoporosis Study (GOS) is a prospective population‐based cohort study involving adults from south‐eastern Australia. An age‐stratified sample of 1540 men was enrolled at GOS‐baseline (2001–2006) using the Australian electoral roll from the Barwon Statistical Division as the sampling frame [23]. Two follow‐up assessments were conducted, at 5 years (2006–2010) and 15 years (2016–2021) (Figure 1). At 2006–2010, 978 (81%) and, at 2016–2021, 629 (61%) returned for review. There were no statistical differences detected in age, mood disorder, education, mobility and measures of obesity and body compositions between those who attended at 2006–2010 and those who did not [19].

The current study was nested within the GOS, including participants with back pain data collected in 2006–2010 (back pain study baseline) and 2016–2021 (back pain study follow‐up). The Human Research Ethics Committee of Barwon Health (Reference Number 0056_E7) approved the study and all participants gave written informed consent [23]. Monash University and Barwon Health have a Research collaboration agreement for the current study.

### 2.2 Low Back Pain and Disability

Back pain and disability in the past 6 months were evaluated using the Graded Chronic Pain Scale (GCPS), a validated questionnaire for evaluating severity of chronic pain and related disability [24, 25]. This includes three questions assessing pain intensity and four questions to assess disability status. Based on pain and/or disability status, participants were categorised into five groups at baseline and follow‐up [25]. Those with no pain and no disability (Grade 0) and low‐intensity pain and low‐disability (Grade 1) were combined and compared with those with high‐intensity pain or high‐disability (Grade 2, 3 or 4). Participants with no or low‐intensity pain and no or low‐ disability at baseline were included in the current study (back pain study baseline, Figure 1). According to pain and/or disability status at follow‐up, participants were categorised as (1) having no or low‐intensity pain and no or low‐disability or (2) incident high‐intensity pain and/or high disability (back pain study follow‐up, Figure 1).

### 2.3 Assessment of Body Composition

Weight measured with participant wearing a hospital gown and height without shoes were measured to the nearest 0.1 cm using a Harpenden stadiometer. Waist circumference was taken between the lower rib and iliac crest for the smallest circumference, and hip circumference was taken in a transverse plane for maximal gluteal circumference, using narrow nonelastic tape. Fat mass (FM) and lean mass (LM) were measured using dual energy X‐ray absorptiometry (DXA; GE Lunar Prodigy, GE Lunar Corp.). BMI (weight in kg/[height in m]^2^), fat‐mass index (FMI = FM/height^2^) and lean mass index (LMI = LM/height^2^) were calculated [19]. All measures were taken at back pain study baseline and prebaseline, 5 years prior. Change in these measures from baseline was calculated by subtracting the prebaseline measure from baseline measure. Thus, positive results indicate gain and negative numbers indicate loss.

### 2.4 Sociodemographic Factors at Baseline

The Hospital Anxiety and Depression Scale (HADS‐D) questionnaire was used to assess depressive affect, with higher scores indicating worse affect (range 0–21) [26]. Information regarding the highest level of education (not completed secondary school vs. above) and mobility status (high mobility [very active or active] vs. low mobility [sedentary, limited, inactive, chair or bedridden or bedfast]) were also collected [19].

### 2.5 Statistical Analysis

Independent sample *t* tests and chi‐square tests were used to compare the baseline characteristics of participants with and without high‐intensity back pain and/or high disability. Binary logistic regression was used to assess the associations with incident high‐intensity pain and/or high disability for all measures of body composition at back pain study baseline, adjusted for age, mobility, education and depression. Additionally, FM and FMI were adjusted for LM and LMI, respectively, and LM and LMI were adjusted for FM and FMI, respectively, as FM and FMI have been suggested to be associated with increased back pain [27] and LM and LMI have been suggested to be protective of back pain [21, 28, 29]. The association between change in body composition in the preceding 5 years from baseline and incident high‐intensity back pain and/or high disability at back pain study follow‐up was also examined using binary logistic regression, adjusted for age, mobility, education, depression, body composition at back pain study baseline and other potential covariates as indicated above. Additionally, exploratory linear relationships between the body composition and incident high‐intensity pain and/or high disability were assessed by fitting restricted cubic splines in the regression model after adjustment, with four knots [30, 31]. The knots were placed at their default position. Figures displaying the splines were demonstrated and interpreted qualitatively. An exploratory sub group analysis based on cut‐off age 60 years [32] and above was performed as measures of body composition change with age [33], and interaction between body composition and age group was tested. We also examined the presence of multicollinearity in the model by examining variance inflation factors (VIF) using multiple linear regression analysis [34]. By convention, if the VIF is more than 5 [35], this suggests the presence of multicollinearity.

All statistical analyses were performed using IBM SPSS Statistics (v 27.0) and nonlinear relationships were performed using STATA (v 16.0). A *p* value less than 0.05 was considered statistically significant.

## 3 Results

At back pain study baseline, of the 831 participants attending, 695 had no or low‐intensity pain and no or low‐disability and with obesity and body composition data available. Men who did not participate in the back study in 2006–2010 (*n* = 147) were not significantly different from those who provided baseline data (*n* = 831). (Table S1). At back pain study follow‐up, 433 participants (62.3%) provided both pain and disability data with two additional participants providing only pain data (Figure 1). Men who completed follow‐up were younger, more likely to have completed secondary school, had lesser depressive features and higher mobility, higher weight, LM and LMI, and lower waist circumference compared to those who did not complete follow‐up (Table S2). Among those who completed the study, 37 (8.5%) developed high‐intensity pain and/or high‐disability, 33 (7.6%) developed high‐intensity pain and 14 (3.2%) developed high‐disability.

The baseline back pain study characteristics of men who developed high‐intensity pain and/or high‐disability were compared with those who remained with no to low pain and disability (Table 1). Men who developed high‐intensity pain and/or high‐disability were more depressed (mean difference [MD] −1.4 95%, CI −2.4, −0.4) and had lower mobility (32.4% vs. 18.2%, *p* = 0.04), compared to those in no or low‐intensity pain and no or low‐disability group. No significant differences in body composition were detected between the two groups. Similar results were observed for the comparisons between men with no or low‐intensity pain and those with high‐intensity pain, and between men with no or low‐disability and those with high‐disability, with the exception of mobility not being significantly different in men with high disability (Table 1).

**TABLE 1:** Comparison between men with no or low‐intensity pain and/or disability and those who developed high‐intensity pain and/or disability at 2016–2021.

Data collected at back pain study baseline (2006–2010)	Pain and/or disability^a^			Pain^b^			Disability^a^		
	No or low *n* = 396	High *n* = 37	*p*	No or low *n* = 402	High *n* = 33	*p*	No or low *n* = 419	High *n* = 14	*p*
Age ^c^ , years	54.1 (14.1)	56.3 (13.8)	0.37	54.2 (14.1)	56.2 (13.7)	0.42	54.2 (14.2)	55.9 (10.9)	0.67
Depression ^c^	2.4 (2.2)	3.7 (2.9)	0.01	2.4 (2.2)	3.7 (2.9)	0.02	2.4 (2.2)	4.6 (2.9)	0.02
Low mobility ^d^	72 (18.2%)	12 (32.4%)	0.04	73 (18.2%)	11 (33.3%)	0.04	79 (18.9%)	5 (35.7%)	0.12
Not completed secondary school ^d^	154 (39.7%)	16 (43.2%)	0.67	156 (39.6%)	16 (48.5%)	0.32	165 (40.1%)	5 (35.7%)	0.74
Measures of body composition									
Weight ^c^, ^e^ , kg	85 (13.4)	82.1 (14.6)	0.22	84.9 (13.4)	82.9 (15.3)	0.42	84.8 (13.5)	84.1 (15.8)	0.86
BMI ^c^, ^e^ (kg/m ^2^ )	27.3 (3.9)	26.6 (4.1)	0.29	27.3 (3.9)	26.9 (4.3)	0.54	27.3 (3.9)	27.2 (4.8)	0.96
WC ^c^, ^f^ , cm	96.7 (11.2)	95.2 (10.5)	0.45	96.7 (11.1)	95.6 (11.1)	0.59	96.6 (11.2)	96.3 (10)	0.92
HC ^c^, ^f^ , cm	101.7 (8.8)	100.5 (8.5)	0.40	101.7 (8.8)	100.8 (8.9)	0.59	101.6 (8.8)	102.0 (8.9)	0.86
FM ^c^, ^g^ , kg	23.0 (8.4)	21.9 (9.6)	0.46	22.9 (8.4)	22.4 (9.7)	0.73	22.9 (8.4)	24.0 (11.3)	0.63
FMI ^c^, ^g^ , kg/m ^2^	7.4 (2.7)	7.1 (3.0)	0.51	7.4 (2.7)	7.3 (3.1)	0.76	7.4 (2.7)	7.8 (3.6)	0.58
LM ^c^, ^g^ , kg	58.9 (6.8)	57.0 (8.7)	0.21	58.8 (6.8)	57.2 (8.4)	0.20	58.8 (6.9)	56.7 (8.2)	0.28
LMI ^c^, ^g^ , kg/m ^2^	19 (1.7)	18.4 (2.2)	0.21	19 (1.7)	18.6 (2.2)	0.21	18.9 (1.7)	18.3 (2.1)	0.20

Abbreviations: BMI, body mass index; FM, fat mass; FMI, Fat Mass Index; HC, hip circumference; LM, lean mass; LMI, Lean Mass Index; WC, waist circumference.

^a^ Data available for 433 participants who provided pain and disability at both time points.

^b^ Data available for 435 for participants who provided pain, but not disability data at both time points.

^c^ Data presented as mean (standard deviation); comparison *p* value for independent *t* test.

^d^ Data presented as number (percentage); comparison *p* value for Chi‐square test.

^e^ Data available for 433 participants.

^f^ Data available for 431 participants.

^g^ Data available for 427 participants.

### 3.1 Association of Body Composition in Baseline (2006–2010) With Incident High‐Intensity Pain and/or High Disability at Follow‐Up (2016–2021)

There were no significant associations between body composition at baseline and incident high‐intensity pain and/or high disability at follow‐up (Table 2). There was a nonlinear relationship between LM and LMI at baseline and incident high‐intensity pain and/or high‐disability at follow‐up (Figure S1).

**TABLE 2:** Association of body composition at baseline (2006–2010) with incident high‐intensity pain and/or high disability at follow up (2016–2021).

Measure (range)	Pain and/or disability OR (95% CI)^a^		Pain OR (95% CI)^a^		Disability OR (95% CI)^a^	
	Model 1	Model 2	Model 1	Model 2	Model 1	Model 2
Weight, kg (55 to 140)	0.98 (0.96, 1.01)	0.98 (0.95–1.01)	0.99 (0.96, 1.02)	0.98 (0.96–1.01)	1.0 (0.96, 1.04)	0.99 (0.95, 1.04)
BMI, kg/m ^2^ (18.3 to 42.8)	0.95 (0.87–1.04)	0.92 (0.84–1.02)	0.97 (0.88–1.07)	0.94 (0.85–1.04)	1.0 (0.87–1.14)	0.98 (0.85, 1.14)
WC, cm (70 to132)	0.99 (0.96, 1.02)	0.97 (0.94,1.01)	0.99 (0.96–1.02)	0.98 (0.94,1.01)	1.0 (0.95–1.05)	0.99 (0.94, 1.04)
HC, cm (74 to134)	0.98 (0.95–1.02)	0.97 (0.93–1.01)	0.99 (0.95,1.03)	0.97 (0.93, 1.02)	1.01 (0.95–1.07)	1.00 (0.94, 1.07)
FM ^2^ , kg (2.27 to 52.2)	0.99 (0.95–1.03)	0.98 (0.93, 1.03)	0.99 (0.95, 1.04)	0.99 (0.94, 1.04)	1.02 (0.96–1.08)	1.02 (0.95, 1.10)
FMI ^3^ , kg/m ^2^ (0.8 to 16.9)	0.96 (0.85–1.09)	0.94 (0.81, 1.09)	0.98 (0.86, 1.12)	0.95 (0.82, 1.11)	1.06 (0.87–1.28)	1.09 (0.86, 1.37)
LM ^4^ , kg 39.6 to 82.30	0.96 (0.92–1.01)	0.97 (0.92, 1.02)	0.97 (0.92, 1.02)	0.97 (0.92, 1.03)	0.96 (0.89–1.04)	0.95 (0.86, 1.04)
LMI ^5^ kg/m ^2^ (14.6 to 26.5)	0.85 (0.67–1.04)	0.87 (0.70–1.07)	0.87 (0.70–1.08)	0.88 (0.71, 1.10)	0.87 (0.58–1.12)	0.80 (0.57, 1.12)

*Note:* Model 1 = unadjusted; model 2 = adjusted for age, depression, completed secondary school or lower level, low‐mobility and another factor as indicated (^2^LM, ^3^LMI, ^4^FM and ^5^FMI).

Abbreviations: BMI, body mass index; FM, fat mass; FMI, Fat Mass Index; HC, hip circumference; LM, lean mass; LMI, Lean Mass Index; WC, waist circumference.

^a^ Odds ratio and (95% Confidence Interval).

### 3.2 Association of Body Composition at Baseline (2006–2010) With Incident High‐Intensity Pain and/or High Disability at Follow‐Up (2016–2021), Stratified by Age Group

The associations between body composition at baseline and incident high‐intensity pain and/or high disability at follow‐up were examined, stratified by median age (Table 3). An increase in LM or LMI was associated with decreased likelihood of developing high‐intensity pain and/or high disability in men aged ≥60 years, with no significant association seen in men aged <60 years (*p* for interaction = 0.03 for LM and <0.001 for LMI). No significant associations were observed for other body composition measures. A significant association for LM or LMI was observed for incident high‐intensity pain but not with high disability in men aged ≥60 years (Table S3).

**TABLE 3:** Association between body composition at baseline (2006–2010) and incident high‐intensity pain and/or high‐disability 10 years later at follow up (2016–2021), stratified by age group.

	<60 years No‐low = 257 High = 24	≥60 years No‐low = 147 High = 13	<60 years No‐low = 257 High = 24	≥60 years No‐low = 147 High = 13	
	Model 1		Model 2		
	OR (95% CI)^a^		OR (95% CI)^a^		*p* ^b^
Weight	0.98 (0.95, 1.02)	0.98 (0.94, 1.03)	0.98 (0.95, 1.01)	0.98 (0.93, 1.03)	0.9
BMI, kg/m ^2^	0.97 (0.87, 1.08)	0.91 (0.76–1.08)	0.96 (0.86, 1.08)	0.86 (0.71, 1.03)	0.4
WC, cm	0.99 (0.96, 1.03)	0.98 (0.92–1.04)	0.99 (0.95, 1.03)	0.95 (0.89, 1.02)	0.5
HC cm	0.98 (0.94, 1.03)	0.98 (0.91–1.06)	0.98 (0.94, 1.04)	0.96 (0.88, 1.04)	0.9
FM ^c^ , kg	0.97 (0.92, 1.02)	1.02 (0.95–1.09)	0.96 (0.91, 1.02)	1.05 (0.96, 1.15)	0.3
FMI ^d^ , (kg/m ^2^ )	0.93 (0.79, 1.09)	1.03 (0.83–1.28)	0.88 (0.73, 1.05)	1.21 (0.82, 1.61)	0.4
LM ^e^ (kg)	0.99 (0.94, 1.05)	0.88 (0.80–0.97)	1.01 (0.94, 1.07)	0.86 (0.76, 0.97)	0.03
LMI ^f^ (kg/m ^2^ )	1.07 (0.85, 1.35)	0.45 (0.29–0.72)	1.14 (0.88, 1.47)	0.36 (0.20, 0.65)	<0.001

*Note:* Model 1 = unadjusted; Model 2 = adjusted for depression, not completed secondary school, low‐mobility and another factor as indicated (^c^LM, ^d^LMI, ^e^FM and ^f^FMI).

Abbreviations: BMI, body mass index; FM, fat mass; FMI, Fat Mass Index; HC, hip circumference; LM, lean mass; LM, Lean Mass Index; WC, waist circumference.

^a^ Odds ratio and (95% confidence interval).

^b^ *p* value for interaction between body composition and age group in adjusted analysis (Model 2).

### 3.3 Association of Change in Body Composition Between Prebaseline (2001–2006) and Baseline (2006–2010) With Incident High‐Intensity Pain and/or High Disability at Follow‐Up (2016–2021)

The associations between change in body composition between pre‐baseline and baseline, and incident high‐intensity pain and/or high disability at follow‐up were examined (Table 4). Incident high‐intensity pain and/or high disability and high‐intensity pain were not associated with change in any measures of body composition. A single unit increase in BMI, which is equivalent to weight gain of 3.1 kg, in the 5 years preceding baseline was associated with 1.63 times incidence of high disability in 2016–2021 [OR 1.63, 95% CI 1.06, 2.51].

**TABLE 4:** Association of change in body composition between prebaseline (2001–2006) and baseline (2006–2010) with incident high‐intensity pain and/or high disability at follow‐up (2016–2021).

	Pain and/or disability OR (95% CI)^a^		Pain OR (95% CI)^a^		Disability OR (95% CI)^a^	
Measure (range)	Model 1	Model 2	Model 1	Model 2	Model 1	Model 2
Weight, kg (−27.8 to 20.3)	1.04 (0.97, 1.12)	1.07 (0.98, 1.17)	1.03 (0.96, 1.11)	1.05 (0.96, 1.14)	1.10 (0.98, 1.22)	1.14 (0.99–1.32)
BMI, kg/m ^2^ (−8.6 to 6.2)	1.17 (0.94, 1.47)	1.28 (0.98, 1.68)	1.12 (0.89–1.42)	1.18 (0.90, 1.55)	1.44 (1.02, 2.04)	1.63 (1.06–2.51)
WC, cm (−20.5 to 22.0)	1.00 (0.95, 1.06)	1.03 (0.96, 1.09)	0.99 (0.93, 1.04)	0.99 (0.93, 1.07)	1.06 (0.97–1.15)	1.10 (0.99, 1.22)
HC, cm (−22 to 18.5)	1.03 (0.97, 1.09)	1.05 (0.99, 1.12)	1.02 (0.97, 1.08)	1.04 (0.97, 1.11)	1.07 (0.98, 1.17)	1.10 (0.99, 1.21)
FM ^2^ , kg (−22.7 to 17.5)	1.02 (0.94, 1.12)	1.05 (0.95, 1.16)	1.01 (0.92, 1.11)	1.02 (0.92, 1.13)	1.13 (0.98, 1.30)	1.14 (0.97, 1.34)
FMI ^3^ , kg/m ^2^ (−6.9 to 5)	1.09 (0.83, 1.44)	1.17 (0.86, 1.61)	1.04 (0.78, 1.39)	1.07 (0.78, 1.50)	1.50 (0.95, 2.63)	1.53 (0.91, 2.59)
LM ^4^ , kg (−7.9 to 9.3)	1.00 (0.86, 1.16)	1.04 (0.88, 1.23)	0.99 (0.85, 1.16)	1.02 (0.85, 1.21)	0.86 (0.67, 1.11)	0.87 (0.65, 1.16)
LMI ^5^ , kg/m ^2^ (−2.5 to 3.1)	1.12 (0.70, 1.78)	1.35 (0.79, 2.29)	1.01 (0.62, 1.65)	1.14 (0.66, 2.0)	0.82 (0.38, 1.77)	0.97 (0.39, 2.39)

*Note:* Model 1 = unadjusted; Model 2 = adjusted for age, mobility, education, depression, back pain study baseline data and another factor as indicated (change in: ^2^LM, ^3^LMI, ^4^FM,^5^FMI). Change in body composition measures = 2006–2010 measures − 2001–2006 measures.

Abbreviations: BMI, body mass index; FM, fat mass; FMI, Fat Mass Index; HC, hip circumference; LM, lean mass; LM, Lean Mass Index; WC, waist circumference.

^a^ Odds ratio and (95% Confidence Interval).

The linear relationship between change in body composition and incident high‐intensity pain and/or high‐disability was checked, showing nonlinear relationship for change in LM and LMI (Figure S2).

### 3.4 Association of Change in Body Composition Between Prebaseline (2001–2006) and Baseline (2006–2010) With Incident High‐Intensity Pain and/or High Disability at Follow‐Up (2016–2021), Stratified by Age Group

The association between change in measures of body composition in the 5 years preceding baseline with incident high‐intensity pain and/or high‐disability at follow‐up was examined, stratified by median age (Table 5). An increase in BMI by one unit was associated with twofold increase in the likelihood of developing high‐intensity pain and/or high disability in men aged ≥60 years. In those over 60 years of age, an increase in BMI of 1 unit was equivalent to an increase in 3 kg. However, there was no significant interaction.

**TABLE 5:** Association of change in body composition between prebaseline (2001–2006) and baseline (2006–2010) with incident high‐intensity pain and/or high disability at follow‐up (2016–2021), stratified by age group.

	<60 years No‐low = 257 High = 24	≥60 years No‐low = 147 High = 13	<60 years No‐low = 257 High = 24	≥60 years No‐low = 147 High = 13	*p* ^2^
	Model 1		Model 2		
	OR (95% CI)^a^		OR (95% CI)^a^		*p* ^b^
Weight, kg	1.02 (0.93, 1.10)	1.10 (0.96, 1.27)	1.05 (0.95, 1.16)	1.15 (0.97, 1.37)	0.6
BMI, kg/m ^2^	1.05 (0.81, 1.38)	1.52 (0.99, 2.33)	1.23 (0.83, 1.53)	2.06 (1.17, 3.64)	0.3
WC, cm	1.02 (0.95, 1.09)	0.98 (0.89, 1.08)	1.05 (0.97, 1.13)	0.98 (0.87, 1.10)	0.3
HC cm	1.04 (0.97, 1.11)	1.00 (0.91, 1.11)	1.07 (1.0, 1.16)	1.01 (0.90, 1.13)	0.4
FM ^c^ , kg	0.99 (0.90, 1.09)	1.13 (0.95, 1.34)	1.03 (0.92, 1.15)	1.18 (0.96, 1.44)	0.2
FMI ^d^ , (kg/m ^2^ )	0.98 (0.72, 1.34)	1.48 (0.87, 2.53)	1.09 (0.76, 1.57)	1.74 (0.92, 3.29)	0.2
LM ^e^ (kg)	1.08 (0.90, 1.29)	0.87 (0.67, 1.12)	1.14 (0.93, 1.39)	0.84 (0.62, 1.13)	0.05
LMI ^f^ (kg/m ^2^ )	1.21 (0.69, 2.12)	0.95 (0.43, 2.13)	1.31 (0.70, 2.45)	1.42 (0.45, 4.14)	0.3

*Note:* Model 1 = unadjusted; Model 2 = adjusted for mobility, education, depression, GOS 1st follow‐up data and another factor as indicated (change in ^c^LM, ^d^LMI, ^e^FM and ^f^FMI). Change in body composition measures = 2006–2010 measures – 2001–2006 measures.

Abbreviations: BMI, body mass index; FM, fat mass; FMI, Fat Mass Index; HC, hip circumference; LM, lean mass; LM, Lean Mass Index; WC, waist circumference.

^a^ Odds ratio and (95% Confidence Interval).

^b^ *p* value for interaction between groups.

Multicollinearity was not present based on the VIFs, which ranged from 1 to 1.2 (Table S6).

## 4 Discussion

This study observed no association between measures of body composition and incident high‐intensity pain and/or high‐ disability over 10 years in a population‐based sample of men. However, higher LM was protective of developing high‐intensity pain and/or high disability in men aged 60 years and over, especially high‐intensity pain over 10 years. An increase in BMI, equivalent to 3.1‐kg weight gain, over 5 years prior was associated with an increased likelihood of incident high‐disability over the next 10 years. Our results suggest that maintenance of LM in older men could be important to reduce back pain‐related burden in future and indicate that recent weight gain may predict poor prognosis after 10 years in men with no or low back pain and disability.

The relationship between BMI and incident high‐intensity back pain and high disability in community‐based men has not been studied. Of the four studies that have examined the relationship between BMI and any incident back pain in longitudinal studies of community‐based men, only one study by Heuch et al. [15] showed a significant relationship (risk ratio 1.09 [1.03–1.16]) in men without any back pain at study inception [14, 15, 16, 17]. Whereas Heuch et al. [15] examined the association in 8725 men (age range 30–69 years), Shiri et al. [16, 17] included 581 men (age range 24–39 years), and Li et al. [14] included 1201 men (average age 61 years). None of these studies examined intensity of back pain and disability using validated questionnaires. Our study, the first study focusing on high‐intensity back pain and/or high disability over 6 months, did not find any association of BMI with development of high‐intensity back pain and/or high disability after 10 years in men. The current study may have limited power to detect this association due to small study sample (*n* = 441).

Change in obesity has been shown to affect back pain. A previous large study (*n* = 6868), involving mainly women (69.3%), reported that an increase in BMI was associated with an increased risk of back pain [22]. However, back pain was assessed as being present or absent: the severity of pain and associated disability were not examined. We have extended this observation and report an association between an increase in BMI by one unit with incident high disability in men 10 years later. This may be explained by the increase in BMI imposing increased mechanical loading on the spine, reducing blood flow or modifying the connective tissue around the spine, which may not be accommodated by neuromuscular change. This may result in disability that is associated with higher costs compared to pain severity only [6, 13, 36]. Our results suggest that for each 3.1‐kg gain in weight over 5 years, there is a 63% greater risk of high disability, and weight gain approximately 2–3 kg is common over 5 years in men [37]. Therefore, community‐based men with history of recent weight gain are at risk of transitioning to high disability and should be targeted for intervention to prevent weight gain related complications in future.

To our knowledge, this is the first longitudinal population‐based study in men to examine the association between FM and LM using DXA, and their change in the preceding 5 years and incident high‐intensity back pain and/or disability over the next 10 years. Although no significant associations were identified in the whole population, a nonlinear U‐shaped association between LM and LMI with high‐intensity pain and/or high‐disability was observed. As higher FM is associated with high LM, this relationship may further deteriorate back pain with high LM resulting in U‐shape association [38]. However, in older, but not younger men, higher LM was protective against the development of high‐intensity pain and/or high disability. LM decreases with age [39]. Our results are in keeping with previous cross‐sectional and case–control studies that show lower LM is associated with poor back pain‐related outcomes in older age [21, 28, 29]. Age‐related muscle loss or sarcopenia may contribute to back pain through malalignment, increased frailty, risk of fracture and chronic inflammation [29, 40]. This study emphasises the importance of maintaining higher LM in men aged over 60 without significant back pain, to be targeted for early intervention to prevent poor outcomes in the long term. Our study highlights the potential risk of high‐intensity back pain associated with age‐related sarcopenia which warrants further exploration.

This study had several strengths. It is the first study in men to examine the longitudinal associations between measures of body composition and incident high‐intensity pain and/or high disability in a population‐based cohort. This study also examined the impact of change in these measures in preceding years on the risk of incident high‐intensity back pain and/or high disability. Validated measures of back pain, FM, and LM were used [25, 41]. This study had some limitations. There is the possibility of selection bias due to lost to follow‐up (retention rate 62.6%). There were significant differences between those who completed the study and those who did not in terms of having been more likely to have completed secondary school, lesser depressive features and higher mobility, higher weight, LM and LMI, and lower waist circumference. Thus, those completing the study tended to have more healthy characteristics than those lost to follow up. Therefore, our results are most generalisable to a healthy male population, who have less depression, are more educated and have better mobility. It is possible that nondifferential misclassification occurred as data regarding back pain were only available at two‐time points over 10 years, which would have resulted in an underestimation of the magnitude of the observed associations. However, once high‐intensity pain or disability develops, it tends to persist at that higher level of pain/disability, minimising the chance of misclassification [4]. Although this study was able to detect significant relationships between LM and LMI in men 60 years and older, it is possible that it lacked power to detect potentially clinically important relationships for other measures of body composition.

## 5 Conclusion

In men without severe back pain and disability, there was no association between body composition and incident high‐intensity pain and/or disability. However, lean mass was protective of incident high‐intensity pain and/or disability in men aged 60 years and over. Additionally, those who have a recent history of weight gain around 3 kg are more likely to develop high‐disability associated with back pain over the next 10 years. These findings have the potential to help health professionals to identify men at increased risk of transitioning to high‐intensity back pain and/or high disability who could be targeted for early intervention to improve patient outcomes and reduce disease burden in the community.

## Ethics Statement

Ethical approval from the Human Research Ethics Committees of Barwon Health (Reference Number 00/56) and informed consent were obtained from all participants included in the study [23]. A research collaboration agreement between Monash University and Barwon Health was obtained.

## Conflicts of Interest

The authors declare no conflicts of interest.

## Supporting information


**Table S1.** Comparison of participants who provided pain and disability data (included in the analysis) and those who did not provide pain and disability data (missing value) in 2006–2010.
**Table S2.** Comparison of participants in the GOS LBP study who completed follow‐up and those lost to follow up.
**Table S3.** Association of measures of body composition at baseline (2006–2010) with developing high‐intensity pain and/or high disability at follow up (2016–21) based on median age (60 years).
**Table S4.** Comparison of Change in measure of Body composition between pre‐baseline (2001–6) and baseline (2006–10) between men with no or low‐intensity pain and/or disability and those who developed high‐intensity pain and/or disability at follow‐up (2016–2021).
**Table S5.** Association between change in body composition from pre‐baseline (2001–2006) to baseline (2006–2010) with developing high‐intensity pain and/or high disability at follow‐up (2016–2) based on median age.
**Table S6.** Multicollinearity diagnosis for predictive factors.
**Figure S1.** The adjusted association between lean mass (A) or lean mass index (B) at baseline (2006–2010) and the odds of incident high‐intensity back pain and/or high‐disability (log scale) at follow up (2016–2021). The solid line represents the odds of incident high‐intensity back pain and/or high‐disability and the blue zone indicates 95% confidence interval.
**Figure S2.** The adjusted association between preceding change in lean mass (A) or lean mass index (B) and the odds of incident high‐intensity back pain and/or high‐disability (log scale). The solid line represents the odds of incident high‐intensity back pain and/or high‐disability and the blue zone indicates 95% confidence interval.
