# Supplementary material for: Body Composition and Incident High‐Intensity Back Pain and/or High Disability: A 10‐Year Prospective Population‐Based Male Cohort
Source: J Cachexia Sarcopenia Muscle. 2024 Nov 24;16(1):e13641. doi: 10.1002/jcsm.13641 (PMC11670176; doi:10.1002/jcsm.13641)
Supplement: Supplementary file 1 — Table S1. Comparison of participants who provided pain and disability data (included in the analysis) and those who did not provide pain and disability data (missing value) in 2006–2010. Table S2. Comparison of participants in the GOS LBP study who completed follow‐up and those lost to follow up. Table S3. Association of measures of body composition at baseline (2006–2010) with developing high‐intensity pain and/or high disability at follow up (2016–21) based on median age (60 years). Table S4. Comparison of Change in measure of Body composition between pre‐baseline (2001–6) and baseline (2006–10) between men with no or low‐intensity pain and/or disability and those who developed high‐intensity pain and/or disability at follow‐up (2016–2021). Table S5. Association between change in body composition from pre‐baseline (2001–2006) to baseline (2006–2010) with developing high‐intensity pain and/or high disability at follow‐up (2016–2) based on median age. Table S6. Multicollinearity diagnosis for predictive factors. Figure S1. The adjusted association between lean mass (A) or lean mass index (B) at baseline (2006–2010) and the odds of incident high‐intensity back pain and/or high‐disability (log scale) at follow up (2016–2021). The solid line represents the odds of incident high‐intensity back pain and/or high‐disability and the blue zone indicates 95% confidence interval. Figure S2. The adjusted association between preceding change in lean mass (A) or lean mass index (B) and the odds of incident high‐intensity back pain and/or high‐disability (log scale). The solid line represents the odds of incident high‐intensity back pain and/or high‐disability and the blue zone indicates 95% confidence interval. [file JCSM-16-e13641-s001.docx]

**Supplementary Table 1: Comparison of participants who provided pain and disability data (included in the analysis) and those who did not provide pain and disability data (missing value) in 2006-2010**

|  | Completed pain and disability data ^a^  n= 831 | Not completed pain and disability data ^b^  n= 147 | Significance of difference |
| --- | --- | --- | --- |
| Age ^c^, y | 59.4 (16.8) | 60.5 (18.8) | 0.49 |
| Depression ^c^, (total score) | 3.0 (2.6) | 3.0 (2.5) | 0.97 |
| Low mobility ^d^ | 234 (28.3%) | 48 (33.6%) | 0.20 |
| Not completed secondary school ^d^ | 378 (46.2%) | 75 (52.8%) | 0.15 |
| Weight | 84.2 (14.4) | 85.1 (15.3) | 0.60 |
| Body mass index ^c^, kg/m^2^ | 27.4 (4.2) | 27.8 (4.3) | 0.45 |
| Waist circumference ^c^, (cm) | 98.1 (11.6) | 98.6 (11.3) | 0.71 |
| Hip circumference ^c,^ (cm) | 102.4 (9.1) | 101.9 (9.2) | 0.60 |
| Fat Mass ^c^, (kg) | 23.5 (8.6) | 24.0 (8.5) | 0.58 |
| Fat Mass Index ^c^, (kg/m^2^) | 7.7 (2.8) | 7.9 (2.7) | 0.53 |
| Lean Mass ^c^, (kg) | 57.7 (7.2) | 57.6 (7.8) | 0.93 |
| Lean Mass Index ^3^, (kg/m^2^) | 18.8 (1.8) | 18.9 (1.7) | 0.88 |

^a^ Participants who completed the chronic pain grade questionnaire at baseline (2006-2010)

^b^ Participants who did not completed the chronic pain grade questionnaire at baseline (2006-2010)

^c^ Data are presented as mean (standard deviation); comparisons were performed using Independent Sample T-test.

^d^ Data are presented as number (percentage); comparison performed using chi-square test

**Supplementary Table 2: Comparison of participants in the GOS LBP study who completed follow-up and those lost to follow up**

|  | Study population ^a^,  n= 433 | LTFU ^b^,  n= 258 | Significance of difference |
| --- | --- | --- | --- |
| Age ^c^, y | 54.3 (14.1) | 66.8 (18.4) | <0.001 |
| Depression ^c^, (total score) | 2.5 (2.3) | 3.3 (2.3) | <0.001 |
| Low mobility ^d^ | 84 (19.4%) | 86 (33.5%) | <0.001 |
| Not completed secondary school ^d^ | 170 (40%) | 132 (52%) | 0.002 |
| Weigh t^c^, kg | 84.8 (13.6) | 82.1 (15.1) | 0.02 |
| Body mass index ^c^, kg/m^2^ | 27.3 (3.9) | 27.3 (4.4) | 0.87 |
| Waist circumference ^c^, (cm) | 96.6 (11.2) | 98.8 (11.9) | 0.01 |
| Hip circumference ^c,^ (cm) | 101.6 (8.8) | 102.6 (9.2) | 0.17 |
| Fat Mass ^c^, (kg) | 22.9 (8.5) | 23.4 (8.8) | 0.50 |
| Fat Mass Index ^c^, (kg/m^2^) | 7.4 (2.7) | 7.8 (2.9) | 0.07 |
| Lean Mass ^c^, (kg) | 58.7 (7.0) | 55.8 (7.5) | <0.001 |
| Lean Mass Index ^3^, (kg/m^2^) | 18.9 (1.8) | 18.6 (1.9) | <0.001 |

^a^ Study population: participants who completed the chronic pain grade questionnaire at baseline (2006-2010) and follow-up (2016-2021)

^b^ LTFU: Participants who completed the chronic pain grade questionnaire at baseline (2006-2010) but not at follow-up (2016-2021)

^c^ Data are presented as mean (standard deviation); comparisons were performed using Independent Sample T-test.

^d^ Data are presented as number (percentage); comparison performed using chi-square test

**Supplementary Table 3:** **Association of measures of body composition at baseline (2006-2010) with developing high-intensity pain and/or high disability at follow up (2016-21) based on median age (60 years)**

| **Pain** | | | | | |  |
| --- | --- | --- | --- | --- | --- | --- |
|  | Univariate analysis | | Multivariate analysis | | P |  |
|  | <60 years  No-low=266  High=22 | >60 years  No-low=156  High=12 | <60 years  No-low=266  High=22 | >60 years  No-low=156  High=12 |  |  |
| Weight | 0.99  (0.96, 1.02) | 0.99  (0.94, 1.04) | 0.99  (0.95, 1.02) | 0.98  (0.93, 1.03) | 1.0 |  |
| BMI, kg/m^2^ | 0.98  (0.88-1.10) | 0.94  (0.79-1.12) | 0.98  (0.86, 1.11) | 0.88  (0.73, 1.06) | 0.5 |  |
| WC, cm | 0.99 (0.96-1.04) | 0.98  (0.92-1.05) | 0.99  (0.95, 1.04) | 0.95  (0.89, 1.02) | 0.6 |  |
| HC cm | 0.99  (0.94-1.04) | 1.0  (0.92-1.08) | 0.99  (0.93, 1.04) | 0.97  (0.89, 1.05) | 0.9 |  |
| FM ^a^, kg | 0.98  (0.93-1.03) | 1.03  (0.96-1.10) | 0.97  (0.91, 1.03) | 1.05  (0.97, 1.15) | 0.3 |  |
| FMI ^b^, (kg/m^2^) | 0.94  (0.79-1.11) | 1.06  (0.85-1.32) | 0.90  (0.74, 1.08) | 1.21  (0.91, 1.62) | 0.4 |  |
| LM ^c^ (kg) | 1.0  (0.94-1.06) | 0.89  (0.81-0.99) | 1.01  (0.94, 1.08) | 0.87  (0.77, 0.98) | 0.04 |  |
| LMI ^d^ (kg/m^2^) | 1.10  (0.86-1.40) | 0.50  (0.32-0.78) | 1.16  (0.89, 1.51) | 0.40  (0.22, 0.71) | 0.002 |  |
| **Disability** | | | | | |  |
|  | Univariate analysis | | Multivariate analysis | | P |  |
|  | <60 years  No-low=294  High=13 | >60 years  No-low=173  High=6 | <60 years  No-low=294  High=13 | >60 years  No-low=173  High=6 |  |  |
| Weight | 0.99  (0.95, 1.04) | 1.02  (0.94, 1.12) | 0.99  (0.94, 1.04) | 1.02  (0.93, 1.11) | 0.5 |  |
| BMI, kg/m^2^ | 0.98  (0.84-1.15) | 1.10  (0.83-1.45) | 0.98  (0.83, 1.16) | 1.07  (0.80, 1.44) | 0.6 |  |
| WC, cm | 1.0  (0.95-1.06) | 1.03  (0.93-1.15) | 1.00  (0.94, 1.06) | 1.03  (0.91, 1.16) | 0.7 |  |
| HC cm | 1.0  (0.93-1.07) | 1.07  (0.95-1.20) | 1.00  (0.93, 1.08) | 1.05  (0.92, 1.20) | 0.4 |  |
| FM ^a^, kg | 1.00  (0.93-1.07) | 1.10  (0.97-1.24) | 1.00  (0.92, 1.09) | 1.37  (0.95, 2.0) | 0.2 |  |
| FMI ^b^, (kg/m^2^) | 1.01  (0.81-1.26) | 1.35  (0.92-1.98) | 1.02  (0.78, 1.33) | 5.0  (0.82, 30.3) | 0.2 |  |
| LM ^c^ (kg) | 0.97  (0.89-1.06) | 0.85  (0.70-1.03) | 0.97  (0.88, 1.07) | 0.55  (0.27, 1.15) | 0.1 |  |
| LMI ^d^ (kg/m^2^) | 0.91  (0.63-1.29) | 0.47  (0.20-1.07) | 0.92  (0.64, 1.33) | 0.02  (0.0, 1.75) | 0.1 |  |

Data are presented as Odds ratio and (95% Confidence Interval).

BMI, Body mass index; WC, Waist Circumference; HC, Hip Circumference; FM, Fat Mass; FMI, Fat Mass Index; LM, Lean Mass; LM, Lean Mass Index.

Multivariate: adjusted for depression, education and mobility and an additional factor, as indicated (^a^ LM, ^b^ LMI, ^c^ FM, ^d^ FMI).

P for interaction between groups.

**Supplementary Table 4: Comparison of Change in measure of Body composition between pre-baseline (2001-6) and baseline (2006-10) between men with no or low-intensity pain and/or disability and those who developed high-intensity pain and/or disability at follow-up (2016-2021)**

| Change in measure of Body composition between 2001-6 and 2006-10 | Pain and/or disability ^a^ | | | Pain ^b^ | | | Disability ^a^ | | |
| --- | --- | --- | --- | --- | --- | --- | --- | --- | --- |
|  | No or low  n = 396 | High  n = 37 | P | No or low  n = 402 | High  n= 33 | P | No or low  n= 419 | High  n= 14 | P |
| Weight ^c, e^; kg | 1.3 (4.8) | 2.2 (5.5) | 0.31 | 1.3 (4.8) | 2.0 (5.6) | 0.44 | 1.3 (4.9) | 3.4 (4.7) | 0.11 |
| BMI ^c, e^  (kg/m^2^) | 0.4 (1.5) | 0.8 (1.8) | 0.17 | 0.4 (1.5) | 0.7 (1.8) | 0.36 | 0.4 (1.5) | 1.3 (1.6) | 0.045 |
| WC ^c, f^, cm | 0.6 (6.3) | 0.8 (6.1) | 0.90 | 0.7 (6.3) | 0.1 (6.1) | 0.61 | 0.6 (6.4) | 2.7 (3.6) | 0.23 |
| HC ^c, f^, cm | 1.7 (6.4) | 2.9 (7.4) | 0.32 | 1.8 (6.4) | 2.7 (7.7) | 0.44 | 1.7 (6.6) | 4.3 (4.8) | 0.16 |
| FM ^c, g^, kg | 2.5 (3.9) | 2.9 (5.7) | 0.61 | 2.5 (3.9) | 2.7 (5.9) | 0.84 | 2.5 (4.0) | 4.4 (3.5) | 0.11 |
| FMI ^c, g^, kg/m^2^ | 0.8 (1.2) | 1.0 (1.8) | 0.54 | 0.8 (1.2) | 0.9 (1.8) | 0.78 | 0.8 (1.3) | 1.4 (1.2) | 0.09 |
| LM ^c, g^, kg | -0.5 (2.2) | -0.5 (3.1) | 1.0 | -0.5 (2.2) | -0.6 (3.0) | 0.91 | -0.5 (2.3) | -1.3 (2.8) | 0.25 |
| LMI ^c, g^, kg/m^2^ | -0.2 (0.7) | -0.1 (1.0) | 0.64 | -0.2 (0.7) | -0.2 (1.0) | 0.97 | -0.2 (0.7) | -0.3 (0.9) | 0.61 |

^a^ Data available for 433 participants who provided pain and disability at both time points.

^b^ Data available for 435 for participants who provided pain, but not disability data at both time points.

^c^ Data presented as mean (standard deviation); comparison p-value for Independent T-test

^d^ Data presented as number (percentage); comparison p value for Chi-square Test

^e^ Data available for 433 participants

^f^ Data available for 431 participants

^g^ Data available for 427 participants

BMI, Body mass index; WC, Waist Circumference; HC, Hip Circumference; FM, Fat Mass; FMI, Fat Mass Index; LM, Lean Mass; LM, Lean Mass Index.

**Supplementary Table 5: Association between change in body composition from pre-baseline (2001-2006) to baseline (2006-2010) with developing high-intensity pain and/or high disability at follow-up (2016-2) based on median age**

| **Pain** | | | | | |  |
| --- | --- | --- | --- | --- | --- | --- |
| Change in measure of Body composition between 2001-6 and 2006-10 | Univariate analysis  OR (95% CI) ^a^ | | Multivariate analysis  OR (95% CI) ^a^ | | P^b^ |  |
|  | <60 years  No-low=254  High=21 | >60 years  No-low=148  High=12 | <60 years  No-low=254  High=21 | >60 years  No-low=148  High=12 |  |  |
| Weight  kg | 1.01 (0.92, 1.10) | 1.09 (0.95, 1.26) | 1.03  (0.93, 1.15) | 1.13  (0.95, 1.35) | 0.64 |  |
| BMI, kg/m^2^ | 1.03 (0.78, 1.37) | 1.34 (0.87, 2.07) | 1.08  (0.79, 1.49) | 1.68  (0.96, 2.94) | 0.59 |  |
| WC, cm | 1.0 (0.93, 1.07) | 0.96 (0.87, 1.06) | 1.03 (0.94, 1.11) | 0.95 (0.84, 1.07) | 0.23 |  |
| HC cm | 1.03 (0.96, 1.11) | 1.01 (0.91, 1.12) | 1.06 (0.98, 1.15) | 1.01 (0.90, 1.14) | 0.54 |  |
| FM ^c^, kg | 0.98 (0.89, 1.09) | 1.09 (0.91, 1.30) | 1.02 (0.90, 1.15) | 1.11 (0.91, 1.36) | 0.33 |  |
| FMI ^d^, (kg/m^2^) | 0.95 (0.69, 1.33) | 1.31 (0.76, 2.25) | 0.91 (0.71. 1,55) | 1.44 (0.76, 1.25) | 0.36 |  |
| LM ^e^ (kg) | 1.04 (0.86, 1.27) | 0.91 (0.70, 1.19) | 1.09 (0.88, 1.35) | 0.89 (0.65, 1.21) | 0.18 |  |
| LMI ^f^ (kg/m^2^) | 1.12 (0.61, 2.05) | 0.84 (0.37, 1.92) | 1.27 (0.45, 3.60) | 0.95 (0.08, 10.9) | 0.30 |  |
| **Disability** | | | | |  |  |
|  | Univariate analysis  OR (95% CI) ^a^ | | Multivariate analysis  OR (95% CI) ^a^ | |  |  |
|  | <60 years  No-low=264  High=11 | >60 years  No-low=155  High=3 | <60 years  No-low=264  High=11 | >60 years  No-low=155  High=3 |  |  |
| Weight,  kg | 1.06 (0.94, 1.20) | 1.25 (0.95, 1.63) | 1.13(0.97, 1.32) | 1.24  (0.88, 1.75) | 0.55 |  |
| BMI, kg/m^2^ | 1.20 (0.81, 1.77) | 3.02 (1.25, 7.34) | 1.40 (0.86, 2.28) | 3.68 (1.08, 12.62) | 0.13 |  |
| WC,cm | 1.05 (0.95, 1.15) | 1.07 (0.88, 1.30) | 1.13 (1.0, 1.28) | 1.06 (0.86, 1.30) | 0.89 |  |
| HC cm | 1.08 (0.97, 1.19) | 1.03 (0.85, 1.25) | 1.13 (1.0, 1.27) | 0.98 (0.80, 1.20) | 0.53 |  |
| FM ^c^, kg | 1.08 (0.92, 1.26) | 1.36 (0.97, 1.90) | 1.15 (0.95, 1.39) | 1.63 (0.91, 2.93) | 0.32 |  |
| FMI ^d^, (kg/m^2^) | 1.26 (0.76, 2.08) | 3.06 (1.03, 9.12) | 1.56 (0.84, 2.91) | 2.99 (0.75, 11.9) | 0.24 |  |
| LM ^e^ (kg) | 0.96 (0.72, 1.28) | 0.60 (0.37, 0.97) | 1.08 (0.77, 1.50) | 0.43 (0.15, 1.19) | 0.03 |  |
| LMI ^f^ (kg/m^2^) | 0.86 (0.35, 2.08) | 0.62 (0.13, 2.87) | 0.79 (0.52, 1.20) | 0.95 (0.08, 1.27) | 0.32 |  |

^a^ Odds ratio and (95% Confidence Interval).

^b^ P value for interaction between body composition and age group in adjusted analysis (model 2)

Model 1= unadjusted, model 2= adjusted for depression, not completed secondary school, low-mobility and another factor as indicated (^c^ LM, ^d^ LMI, ^e^ FM, ^f^ FMI)

BMI, Body mass index; WC, Waist Circumference; HC, Hip Circumference; FM, Fat Mass; FMI, Fat Mass Index; LM, Lean Mass; LM, Lean Mass Index.

**Supplementary Table 6: Multicollinearity diagnosis for predictive factors**

| Measure | Pain and/or disability | |
| --- | --- | --- |
|  | Model 1  OR (95% CI) ^a^ | VIF ^b^ |
| Weight, kg | 0.98 (0.95-1.01) | 1.0 |
| BMI, kg/m^2^ | 0.92 (0.84-1.02) | 1.1 |
| WC, cm | 0.97 (0.94,1.01) | 1.2 |
| HC, cm | 0.97 (0.93-1.01) | 1.1 |
| FM^2^, kg | 0.98 (0.93, 1.03) | 1.2 |
| FMI^3^, kg/m^2^ | 0.94 (0.81, 1.09) | 1.2 |
| LM^4^, kg | 0.97 (0.92, 1.02) | 1.2 |
| LMI^5^ kg/m^2^ | 0.87 (0.70-1.07) | 1.2 |

^a^Odds ratio and (95% Confidence Interval) based on logistic regression.

^b^Variance inflation factor

BMI, Body mass index; WC, Waist Circumference; HC, Hip Circumference; FM, Fat Mass; FMI, Fat Mass Index; LM, Lean Mass; LM, Lean Mass Index.

Model 1= adjusted for age, depression, completed secondary school or lower level, low-mobility and another factor as indicated (^2^LM, ^3^LMI, ^4^FM, ^5^FMI)

**Supplementary Figure 1**


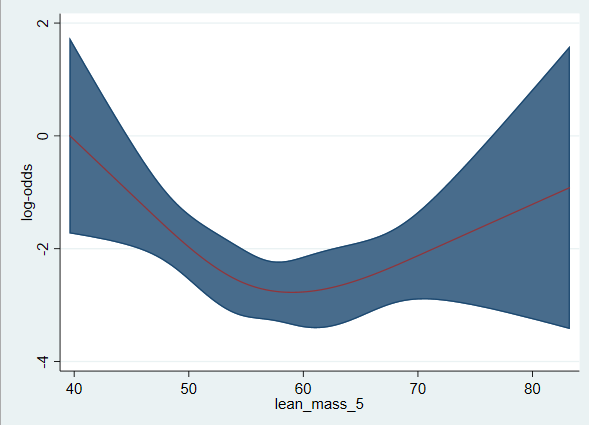

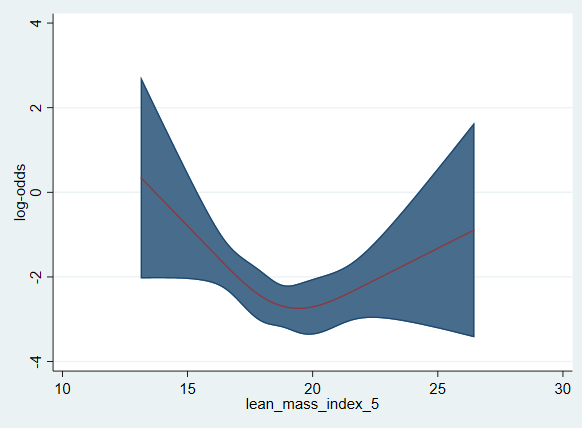


b

a

**Supplementary Figure 1**: The adjusted association between lean mass (A) or lean mass index (B) at baseline (2006-2010) and the odds of incident high-intensity back pain and/or high-disability (log scale) at follow up (2016-2021). The solid line represents the odds of incident high-intensity back pain and/or high-disability and the blue zone indicates 95% confidence interval.

**Supplementary Figure 2**

a


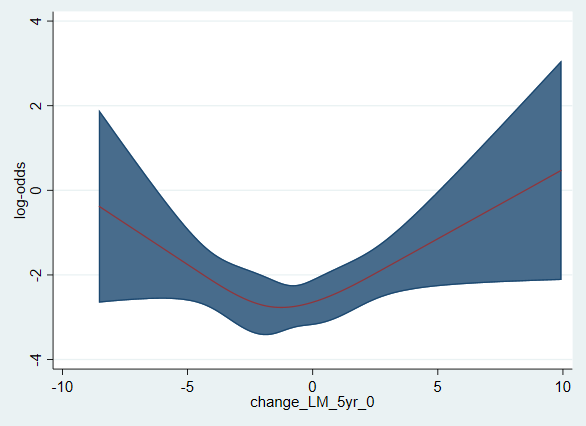

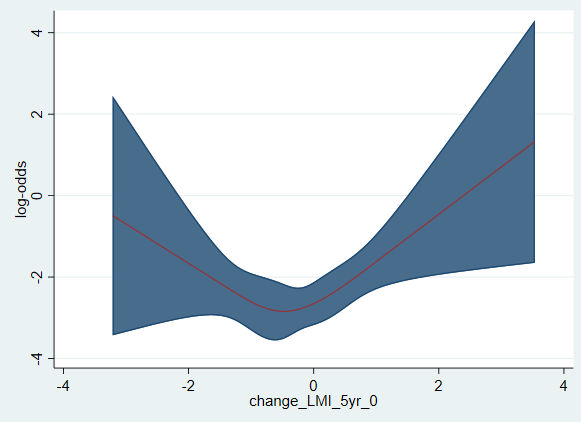


b

**Supplementary Figure 2:** The adjusted association between preceding change in lean mass (A) or lean mass index (B) and the odds of incident high-intensity back pain and/or high-disability (log scale). The solid line represents the odds of incident high-intensity back pain and/or high-disability and the blue zone indicates 95% confidence interval.
